# Risk Factors Affecting Mortality in Asian Hemodialysis Patients: A 15-Year Study From Pakistan

**DOI:** 10.7759/cureus.77328

**Published:** 2025-01-12

**Authors:** Sumbal Nasir Mahmood, Najia Hameed, Humza Kunwer Naveed, Muhammad Fazal Hussain Qureshi, Syeda Hooria Imtiaz, Amna Saleem Rajput

**Affiliations:** 1 Nephrology, Dr. Ziauddin Hospital, Karachi, PAK; 2 Medicine, Aga Khan University Hospital, Karachi, PAK; 3 Nephrology, Ziauddin University, Karachi, PAK; 4 Internal Medicine, Dow University of Health Sciences, Karachi, PAK

**Keywords:** arteriovenous (av) fistula, central venous catheter (cvc), end stage renal disease (esrd), hd ( hemodialysis ), mortality#

## Abstract

Background

End-stage renal disease (ESRD) impacts millions worldwide, with higher mortality rates despite progress in renal replacement therapy. Although numerous risk variables have been recognized, comprehensive long-term data from South Asia remain scarce. Various studies have been done to identify risk factors causing mortality in ESRD patients on hemodialysis (HB), including diabetes, cardiovascular disease, infections, age and dialysis prescription, and its compliance, and efforts to improve the outcome in this vulnerable population. However, the majority of these studies are shorter.

We conducted this study for 15 years to see the effect of various risk factors on mortality and to identify modifiable risk factors to improve survival in this patient population.

Materials and methods

Retrospective cohort research was undertaken at the dialysis unit of a tertiary care hospital, analyzing risk variables and mortality in patients with ESRD undergoing maintenance HD from January 2006 to December 2021. Data were gathered utilizing a pre-structured questionnaire encompassing demographics, comorbidities, dialysis frequency, vascular access type, and survival outcomes. Statistical analysis utilized SPSS (IBM SPSS Statistics for Windows, IBM Corp., Version 25, Armonk, NY), employing chi-square tests for categorical variables and Cox regression for survival analysis, with p < 0.05 deemed significant.

Results

Nine hundred sixty-nine patients were enrolled, 326 were lost to follow, and 84 (13%) expired. The study comprised 54.8% (46) male and 45.2% (38) female, with an average survival period of 28.4 months. The results indicated that thrice-weekly HD had significantly improved survival compared to twice-weekly HD (p < 0.05). The utilization of CVC is associated with an elevated mortality risk (HR = 2.18, p < 0.05) in comparison to AV fistula. Male sex and diabetes were independent predictors of mortality (p < 0.05). The predominant causes of mortality were undetermined (34.5%), infections/sepsis (28.6%), and heart conditions (26.2%).

Conclusion

This 15-year study identifies modifiable risk factors influencing mortality in Pakistani patients with ESRD. Enhancing HD frequency and circumventing CVC utilization may markedly increase survival rates. The results highlight the necessity for timely AV fistula development and adequate dialysis frequency in resource-constrained environments.

## Introduction

End-stage renal disease (ESRD) patients require renal replacement therapy to sustain life through dialysis (hemodialysis or peritoneal) or kidney transplantation. Although the mortality rate for dialysis patients remains higher than the overall population [1], it has significantly reduced over the past few years [2]. Nevertheless, some studies suggest otherwise [3]. Inflammation, disturbances in iron metabolism and erythropoiesis, cardiovascular events, and nutritional status have all been implicated as risk factors for increasing mortality. In addition, using central venous catheters (CVC), hypoalbuminemia, and Kt/V are significantly associated with mortality in various studies.

Since the initiation of maintenance HD, optimal dialysis dosing has always remained controversial. In some studies, the frequency of HD has been reported as an important prognostic factor for patient survival [4]. On the contrary, the impact of long-term dialysis on nutritional status and varying dialysis frequencies has been observed to result in varying complications [5].

In light of literature-based evidence, a minimum single-pool Kt/Vurea of 1.2 corresponding approximately to urea reduction ratio (URR) of 65% per session of hemodialysis (HD), and single pool Kt/V of a minimum of 3.4 has been recommended by Kidney Disease Outcomes Quality Initiative (KDOQI) Guidelines and European Guidelines, respectively, for patients having thrice-weekly HD [6]. However, due to financial constraints and high illiteracy rates, twice-weekly HD is a usual practice in lower-middle-income countries. Once/week HD or "as needed" HD has also been observed in around one-fourth of the patients requiring dialysis [7]. However, due to a lack of formal national registries for ESRD, reliable data on the true incidence and prevalence of ESRD in Pakistan is still lacking; approximately only 10% of ESRD patients undergo dialysis. Many of them do not survive with the highest mortality rate in the initial three months of dialysis [8], either due to inadequate HD frequency or type of vascular access used at the time of initiation of HD or starting dialysis very late when the patient is fully uremic or has uremic complications [9-11]. High mortality has been observed in patients who got dialyzed via CVC, succumbing to death due to catheter-related sepsis [12,13].

We conducted this study to identify the modifiable and non-modifiable risk factors resulting in the cause of death in HD patients, such as the frequency of HD and type of vascular access, besides gender, diabetes, hypertension, and coronary heart disease.

## Materials and methods

A retrospective cohort research was performed to examine data gathered over a 15-year period, from January 2006 to December 2021, at Ziauddin Medical University. The research obtained ethical approval from the Institutional Review Board, assuring adherence to research protocols. The researchers utilized a convenience sample strategy, choosing patients from hospital records based on availability instead of random selection. The study encompassed adult patients aged 18 to 80 years diagnosed with ESRD who died during maintenance HD sessions conducted either biweekly or triweekly. These patients utilized arteriovenous (AV) fistulas or CVCs for vascular access. Patients were eliminated if they were lost to follow-up, did not undergo regular dialysis, or had malignancies, as these factors could independently affect survival results.

Data collection tool and procedure

The data gathering included a pre-structured questionnaire to guarantee uniformity and precision. Data were obtained from the records of 969 patients who satisfied the inclusion criteria. The gathered data encompassed information regarding age, gender, comorbidities, dialysis session frequency, type of vascular access, average survival duration, and cause of death.

Statistical analysis

Statistical analysis was conducted utilizing the Statistical Package for Social Sciences (SPSS) (IBM SPSS Statistics for Windows, IBM Corp., Version 25, Armonk, NY). Descriptive statistics were utilized, summarizing quantitative factors, like age and survival time, as mean values with standard deviations, while qualitative variables, such as gender and type of vascular access, were reported as frequencies and percentages. Inferential statistics utilized the chi-square test to determine connections between variables, with a p-value below 0.05 being statistically significant at a 95% confidence interval.

Cox regression analysis was used to evaluate the association between independent variables and average survival time following the commencement of HD. Furthermore, survival and hazard function patterns were visually represented to facilitate an intuitive comprehension of survival trends and hazard rates over time.

## Results

Nine hundred sixty-nine patients were enrolled in the dialysis unit from January 2006 to December 2021. Of those, 326 patients were lost to follow-up or did not meet inclusion criteria and were excluded. Only 13% (n = 84) of the patients expired, and their data were used for analysis. Demographics are shown in Table 1. The mean age of the study subjects was 54 years, with a standard deviation of 9.2 years; 54.8% (n = 46) of the sample consisted of male gender while 45.2% (n = 38) comprised female gender. Our study patients had an average survival period of 28.4 ± 30.2 months after their first dialysis. Patients undergoing dialysis thrice weekly had greater mean survival time. Diabetes mellitus and hypertension also had a significant relation with mortality, with a p-value of less than 0.05, as shown in Table 1.

**Table 1:** Demographics p < 0.05 indicates statistical significance; *chi-square; n = number of patients; % = percentage.

Comorbid	Male n (%)	Female n (%)	Total	p-value
Diabetes mellitus	40 (87%)	35 (92%)	75 (89.3%)	0.02*
Hypertension	41 (89%)	33 (87%)	74 (88.1%)	0.01*
Ischemic heart disease	21 (46%)	24 (63%)	45 (53.6%)	0.06*
Chronic obstructive pulmonary disease	2 (4.3%)	3 (7.9%)	05 (6%)	0.18*
Chronic liver disease	01 (2.2%)	02 (5.3%)	03 (3.6%)	0.69*
Hyperparathyroidism	01 (2.2%)	01 (2.6%)	01 (2.4%)	0.99*
Route of dialysis				
Arteriovenous fistula (AVF)	23	21	44 (52%)	0.03*
Permacath	23	17	40 (48%)	
Frequency of dialysis				
Twice per week	27	19	46 (54.8%)	0.04*
Thrice per week	18	18	36 (42.9%)	

**Figure 1:**
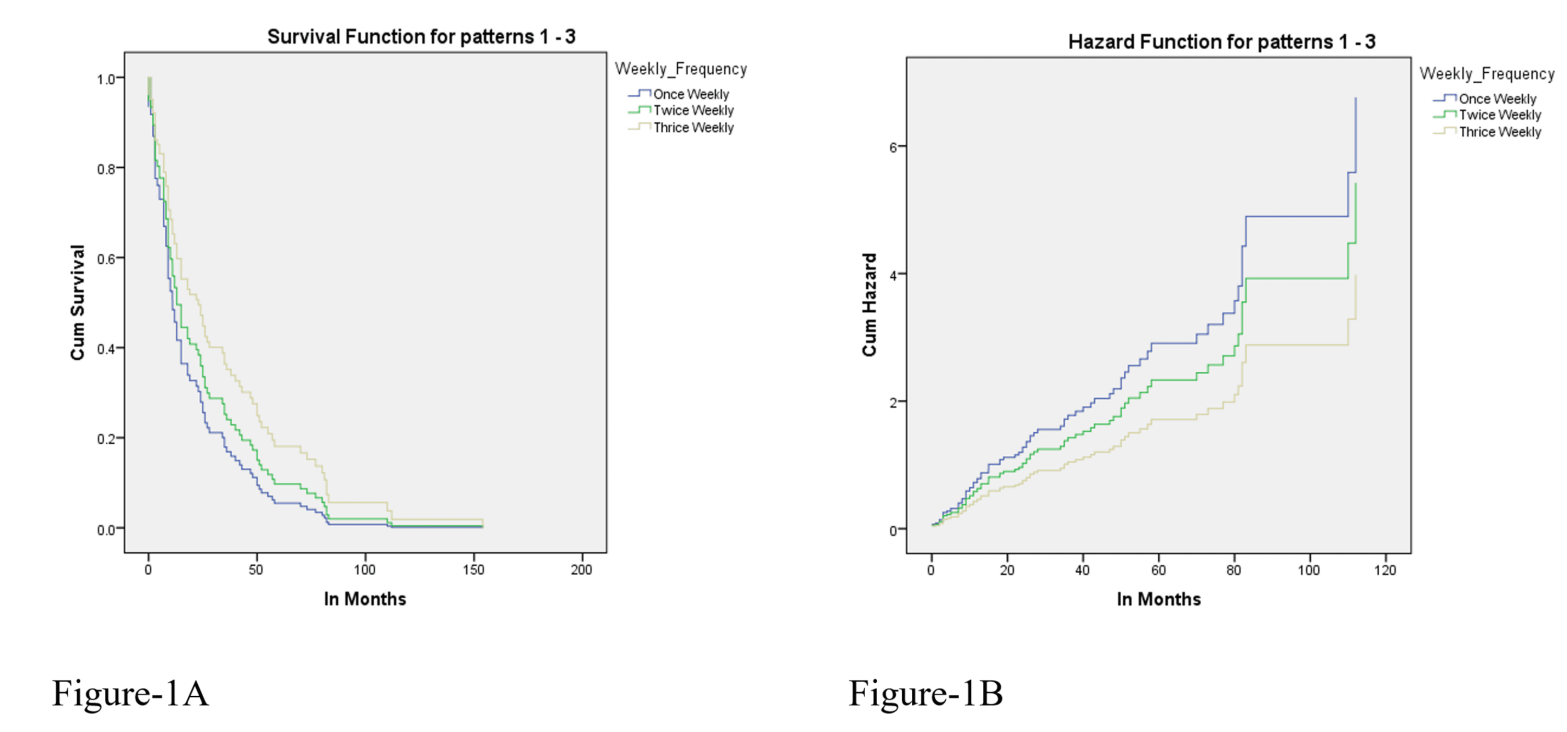
Relationship between dialysis frequency and survival. (A) Kaplan-Meier estimate; (B) Cox proportional hazards model.

Kaplan-Meier survival between the groups is shown in Figure 1.

**Figure 2:**
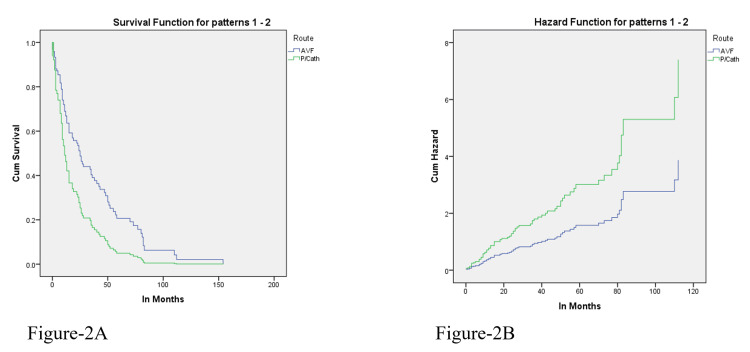
Relationship between vascular access and survival. (A) Kaplan-Meier estimate; (B) Cox proportional hazards model.

When the Cox regression model was applied to compare the average survival duration with AV fistula and CVC use, a significant p-value of p < 0.05 was found. The cumulative hazard for AV fistula was significantly lower than the cumulative hazard of CVC, and the survival function graph showed that cumulative survival for patients with CVC was low (0.08), while for AV fistula was high (0.3), as shown in Figure 2.

**Figure 3:**
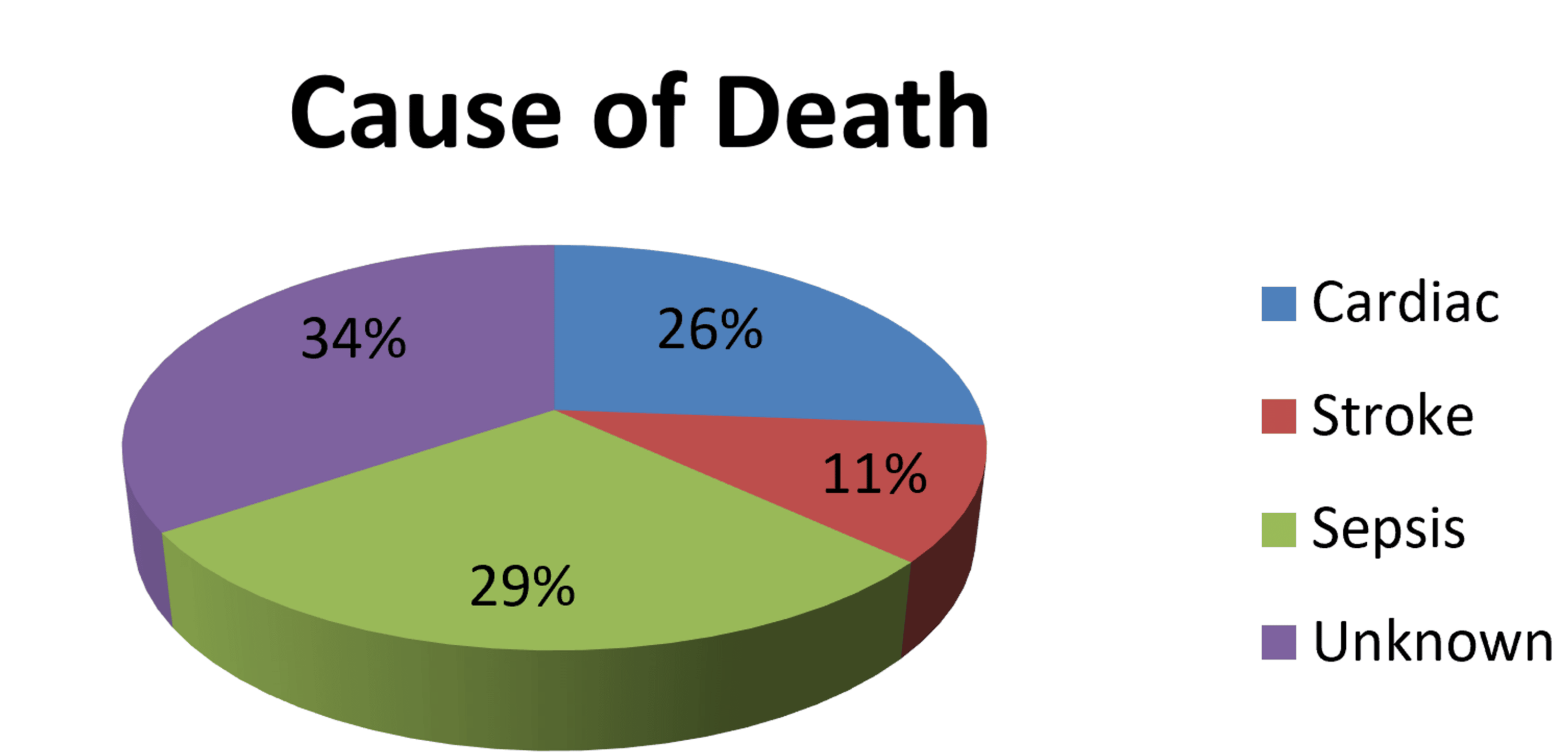
Pie chart showing causes of death

Cause of death was divided into four major categories: cardiac causes, stroke, infection/sepsis, and unknown cause; 34.5% (n = 29) patients died of unknown causes at their residence, 28.6% (n = 24) patients died of infection/sepsis, out of which 11.9% (n = 10) patients died of COVID, 26.2% (n = 22) died of cardiac causes and 10.7% (n = 9) of patients died because of stroke out of which three had an intracranial bleed as shown in a pie chart (Figure 3).

## Discussion

To date, this is the first study showing the impact of dialysis frequency on survival rates among patients undergoing HD in Pakistan.

Our patients had an average survival period of 28.4 ± 30.2 months. Joly et al. also reported a median survival of 28.9 months in a comparison study among patients opting for dialysis [14]. The survival period observed in our study is greater than the estimated mean survival time found in patients undergoing HD in India, which was 19.2 months, with a mortality rate of 19.8% by Chandrashekar et al. [15].

This study also showed that 42.9% of patients (36 patients) underwent thrice/week HD, and 54.8% (46 patients) had twice/week HD only. Better survival was observed in those undergoing HD three times per week than in those who were dialyzed twice weekly. Sun et al. also reported a significant association of HD frequency with the mortality rate in patients undergoing HD. They demonstrated 4.26 times less survival in those dialyzed twice/week vs. those dialyzed thrice weekly [16]. Our study results contrast the findings reported by the Shanghai Renal Registry, which found similar survival rates of both twice and thrice dialyzed patients; however, analysis is confined because of a lack of data on residual renal functions [17].

AV fistula has been recommended as the safest vascular access for better blood flow rates and reduced access-related infections, resulting in better survival than central venous access [18-21]. This study also demonstrated a significantly high survival duration in patients who underwent HD via AV fistula. In contrast, in patients where CVC was used as vascular access, remarkably lower survival duration was observed, and sepsis was attributed to overall mortality in 28.6% (n = 24) of patients. This was also observed in Dialysis Outcomes and Practice Patterns Study (DOPPS) data, showing an increased relative risk of death of 1.31 for patients dialyzed via a CVC. A recent USRDS study also found a relative risk of 2.18 with using a catheter compared to AV fistula usage [18]. Similarly, in a study from Canada, a six times higher risk of mortality was seen with the use of a catheter in comparison to the combined use of AV fistula or AV graft [22]. Kim et al. also reported better survival in patients with AV fistula in comparison to other access [23].

Cardiovascular disease accounted for almost a quarter of deaths in this population, a cause that is the major contributor to mortality in ESRD patients. However, a study by Shastri et al. showed that non-cardiac causes of death are equally responsible for mortality in HD patients, where sudden cardiac death accounted for 22% of all fatal events and non-cardiac death caused 78% [24]. These results are almost similar to our study, where infections/sepsis and neurological events accounted for almost 40% of deaths.

Limitations

Our study has a few limitations. It is a single-center study with a small sample size, so more studies should be conducted involving multiple dialysis units to improve dialysis adequacy and increase survival in the Asian ESRD population. Drug compliance was not assessed via a structured questionnaire, limiting the study as non-adherence can increase morbidity and mortality in the studied population. Hence, the study also raises the need for the implementation of validated and well-described questionnaires in our dialysis setups, along with the calculation of pick-up and refill rates, to improve the survival rate in the ESRD population.

## Conclusions

In conclusion, our study identifies modifiable risk factors that could significantly improve the morbidity and mortality rates in ESRD patients, notably sepsis, frequency of dialysis, and strict blood pressure control that may reduce the incidence of CNS events. It emphasizes the need to optimize the dialysis practice to improve the survival of patients on HD. It highlights the importance of timely instillation of AV fistula in patients with CKD stage V and the implementation of pre-dialysis renal care, focusing on the optimal start of dialysis with cannulate AV fistula and avoiding the use of CVC. Also, it is worth noting that according to a cross-sectional study conducted a few years back, for a population of 160 million, there were only 80 nephrologists in Pakistan who were formally trained, and, at present, many dialysis centers are working autonomously where dialysis sessions are shortened so that extra patients can be accommodated. Integration of a robust system comprising trained nephrologists and dedicated dialysis technicians and financing of health services should be promoted.
